# Folate alleviated skin inflammation and fibrosis resulting from impaired homocysteine metabolism

**DOI:** 10.1016/j.redox.2025.103501

**Published:** 2025-01-21

**Authors:** Jiefeng Huang, Wuyan Lu, Shenli Zhao, Zixin Cai, Linxiao Li, Zihao Hu, Yu Jiang, Jinyi Deng, Yiming Tang, Chenzhang Shi, Chen Wang, Guangpeng Liu, Shuaijun Li

**Affiliations:** a Department of Plastic Surgery, Shanghai Tenth People's Hospital, Tongji University School of Medicine, Shanghai, 200072, China; b The First Affiliated Hospital of Wannan Medical College, Yijishan Hospital of Wannan Medical College, Anhui, 241001, China; c Department of Gastrointestinal Surgery, Shanghai Tenth People's Hospital, Tongji University School of Medicine, Shanghai, 200072, China; d Department of Plastic Surgery, Shanghai Ninth People's Hospital, Shanghai Jiao Tong University School of Medicine, Shanghai, 200011, China

**Keywords:** Skin fibrosis, Inflammation, Homocysteine, Folate, JAK2/STAT3 signaling pathway

## Abstract

Skin fibrosis, characterized by uncontrolled secretion of extracellular matrix (ECM) proteins such as collagen, can lead to excessive scarring and compromised tissue function. Despite the widespread occurrence of fibrotic diseases, effective therapies are lacking. Recent clinical studies have demonstrated a positive correlation between serum homocysteine (Hcy) levels and the severity of systemic sclerosis. However, it remains unclear whether Hcy accumulation plays a pathogenic role in skin fibrosis. Here, we report that Hcy metabolism in fibroblasts plays a crucial role in regulating the pathogenesis of skin fibrosis. Fibrotic skin fibroblasts exhibited elevated levels of Hcy due to the downregulation of catabolism genes *CBS* and *MTR*. Experimental skin fibrosis was induced and exacerbated in mouse skin fibroblasts and tissues through adenoviral knockdown of *Cbs* or *Mtr*, whereas overexpression of these catabolic genes mitigated the pathogenesis. Furthermore, exogenous Hcy supplementation induced and aggravated the expression of inflammatory and fibrotic genes, promoting both spontaneous and BLM-induced skin fibrosis. Notably, folate administration enhanced Hcy catabolism and ameliorated skin inflammation and fibrosis by inhibiting JAK2/STAT3 signaling pathway. Collectively, these results indicate that skin fibrosis is associated with Hcy metabolic disorders and suggest that targeting Hcy metabolism or supplementing folate may provide a novel strategy for skin fibrosis.

## Highlights

• Hcy accumulation due to impaired catabolism contributes to fibrotic skin pathology.
• Targeted regulation of Hcy-metabolizing enzymes CBS and MTR is critical in fibrosis.
• Hcy supplementation induces and exacerbates skin inflammation and fibrosis.
• Folate treatment reverses inflammation and fibrotic markers by catabolizing Hcy.
• The JAK2/STAT3 signaling pathway is a central mediator of Hcy-induced skin fibrosis.

## Graphical abstract

**Figure:**
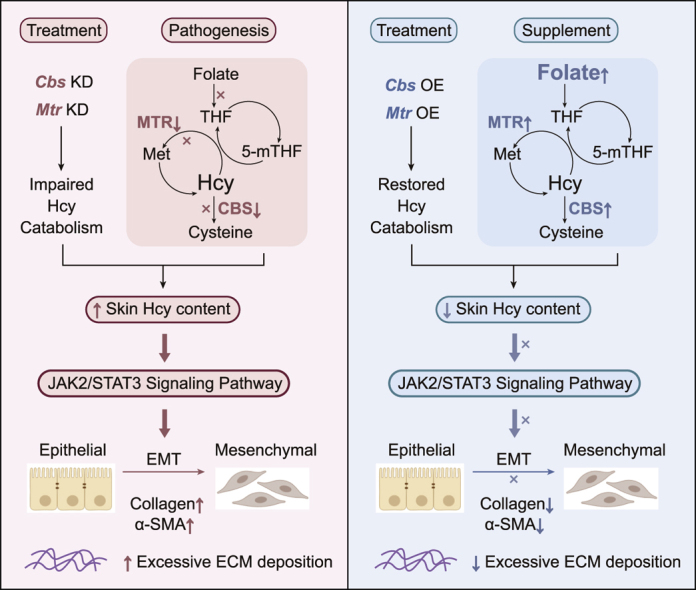

Model depicting the mechanisms of homocysteine accumulation in exacerbating skin fibrosis. In skin fibrotic conditions, Hcy catabolism was impaired, concerning the decrease of CBS, MTR, MTRR, and MTHFR, leading to elevated levels of Hcy. Consequently, Hcy accumulation enhanced JAK2/STAT3 pathway activation, thus promoting epithelial mesenchymal transition and exacerbating skin fibrosis. With folate supplement, the expression of Hcy catabolism genes were reversed, and the content of Hcy was diminished. Folate supplementation mitigated JAK2/STAT3 activation, providing protective effects against skin fibrosis.

## 1 Introduction

Skin fibrosis is a fibro-proliferative disorder affecting the dermis, including scleroderma, hypertrophic scars (HS), keloids, and graft-versus-host disease [1,2]. This disorder occurs following injury to the reticular dermis and extends outward beyond the original wound, exhibiting features comparable to tumors [3]. Consequently, it is often referred to as an over-healing fibroproliferative disease [4]. Although treatments such as surgery combined with 5-fluorouracil chemotherapy or triamcinolone injections can delay disease progression, the recurrence rate remains high [5]. Patients frequently suffer from long-term symptoms like itching and pain. Moreover, uncontrolled skin fibrosis can lead to significant functional impairments, including joint contractures, irreversible structural deformities, and growth retardation in children [6]. Despite its widespread impact, the etiology of skin fibrosis remains elusive, and there are currently no fully curative treatments available.

During skin fibrosis, transforming growth factor β1 (TGF-β1) is activated, promoting dermal fibroblasts to become myofibroblasts [7]. These myofibroblasts express α-smooth muscle actin (α-SMA), proliferate, migrate to the wound, and deposit complex extracellular matrix (ECM) [7,8]. Progressive fibrotic pathology is also characterized by the triggering of inflammatory cells and the overexpression of pro-inflammatory factors such as interleukin-1β (IL-1β), IL-6, IL-17, and tumor necrosis factor-α (TNF-α) [9]. Interestingly, both IL-6 and its second messengers, Janus kinase 2 (JAK2) and signal transducer and activator of transcription 3 (STAT3), are elevated in keloids [10]. The IL-6/JAK/STAT3 pathway has been reported as a key mechanism in epithelial-mesenchymal transition (EMT) pathogenesis in other organ systems [11]. Previous study showed that IL-6-dependent STAT3 activation positively regulates TGF-β1-induced EMT and invasion in hepatocellular carcinoma [12]. Additionally, IL-6 promotes EMT by activating the JAK/STAT pathway, leading to fibrotic injury of the peritoneal membrane [13]. Therefore, identifying the active mechanisms of JAK/STAT underlying skin fibrosis may shed light on novel therapeutic strategies.

Homocysteine (Hcy) is a non-essential sulfur-containing amino acid, which is formed via the classical remethylation and transsulfuration pathways [14]. Hyperhomocysteinemia (HHcy) is a metabolic disorder caused by improper removal and/or accumulation of Hcy most commonly arising from low dietary intake of folate or vitamin B12, or decreases in 5-methyltetrahydrofolate homocysteine methyltransferase (*Mtr*) and cystathionine β-synthase (*Cbs*) genes [15]. In the transsulfuration pathway, CBS is responsible for the pyridoxal phosphate-dependent conversion of Hcy to cystathionine [16]. MTR also plays a role in Hcy catabolism by catalyzing the regeneration of Methionine (Met) from Hcy [16]. Elevated levels of Hcy have been associated with cytotoxic, proinflammatory, and proatherogenic effects, which are linked to cardiovascular disease and diabetes [17]. Recent studies have demonstrated that serum Hcy levels are elevated in systemic sclerosis (SSc) patients [18]. Additionally, Madhulika Tripathi et al. have shown a significant positive correlation between Hcy levels and the progression of non-alcoholic steatohepatitis (NASH) [15]. Furthermore, knockdown of *Cbs* increased liver fibrosis and dietary supplementation with vitamin B12 potentially alleviate NASH through the modulation of Hcy metabolism [15]. However, it remains unclear whether Hcy plays a pathogenic role in skin fibrosis and whether folate supplementation can alleviate skin fibrosis.

In this study, we examined the role of Hcy in fibrotic skin in both mouse models and human patients. Our findings revealed that Hcy accumulates in fibrotic skin due to decreased levels of CBS and MTR. We also validated a significant positive correlation between Hcy levels and the severity of skin fibrosis through both in vivo and in vitro investigations. Additionally, we discovered that folate supplementation can attenuate skin fibrosis by inhibiting the JAK2/STAT3 signaling pathway.

## 2 Materials and methods

### 2.1 Data preparation and Identification of differentially expressed genes

We searched and downloaded mRNA expression data of fibrotic skin pathology from the Gene Expression Omnibus (GEO) database (https://www.ncbi.nlm.nih.gov/geo/) through the keyword “skin fibrosis”. Keloids (GSE145725, GSE32413, GSE44270, GSE76807, GSE188952, GSE212954, GSE246562), bleomycin (BLM)-induced fibrotic mice skin (GSE71998, GSE226331), and transforming growth factor-beta 1 (TGF-β1)-treated fibroblasts (GSE99999, GSE264288) analyses were obtained and conducted. The list of upregulated and downregulated genes in each dataset was sorted by log2FC (adjusted P value < 0.05 and |Log2FC| ≥ 1.5). Heatmap and UpsetR visualizations were plotted by https://www.bioinformatics.com.cn.

### 2.2 Enrichment analysis

Through the Database for Annotation Visualization and Integrated Discovery (DAVlD) tools (https://david.ncifcrf.gov), we carried out enrichment analyses, through the Gene Ontology (GO) and Kyoto Encyclopedia of Genes and Genomes (KEGG) databases. GO is utilized in functional annotation and enrichment analysis, containing biological process (BP), molecular function (MF), and cellular component (CC). KEGG aims to collect large numbers of data about molecular-level information, biological pathways and chemical substances generated by high-throughput experimental technologies. Only p values < 0.05 were considered statistically significant. To further gain insight into the biological processes and predict potential signaling pathways, Gene Set Enrichment Analysis (GSEA) analyses were obtained (https://www.gsea-msigdb.org/gsea/index.jsp). The R package “clusterprofiler” was employed for gene set enrichment analysis and visualization of signaling pathways.

### 2.3 Construction of the protein-protein interaction (PPI) network

Utilizing the Search Tool for Retrieval of Interacting Genes (STRING) database (https://string-db.org/), homo sapiens was the only species considered for the creation of a PPI network. Through mutual mapping of disease targets and drug targets, the PPI network was constructed, incorporating the mapping results into the STRING database. Junction sizes and colors were the main factors employed by the PPI network to assess the protein interaction levels.

### 2.4 Human clinical samples

The collection of discarded human tissues was approved by Shanghai Ninth People's Hospital. Written informed consent was provided by all patients involved in this study. The study design and conduct complied with all relevant regulations regarding the use of human study participants and was conducted in accordance with the criteria set by the Declaration of Helsinki. The characteristics of each normal and hyperplastic scar tissue were substantiated based on clinical diagnosis and pathology, with specific characteristics presented in Table. S1. Scar tissues were obtained from 15 hyperplastic scar patients, while normal skin tissues were obtained from 15 healthy individuals. Following the isolation, the specimens were processed for hydroxyproline and Hcy concentration detection, accompanied by quantitative real-time polymerase chain reaction (qPCR). The remaining tissues were sectioned and then subjected to histological examination. Furthermore, the tissues were also collected for primary fibroblast culture.

### 2.5 Animal experiment

10-week-old male ICR mice (weighing 30–35g) were purchased from Shanghai SLAC Laboratory Animal Co.Ltd., and maintained under specific pathogen-free (SPF) conditions in animal center of Tongji University School of Medicine. Mice were housed under ambient temperature of 24 ± 2 °C, circulating air, constant humidity of 50 ± 10 % and a 12 h:12 h light/dark cycle. The animal sample size for each experiment was selected based on previous, well-characterized studies. The experimental procedures involving the capture, holding, anesthesia, surgery and euthanasia of mice were performed in accordance with the Guidelines for the Care and Use of Laboratory Animals and approved by the Animal Care and Experiment Committee of Tongji University.

Mice were randomized into 16 groups. In knockdown and overexpression models, adenovirus-encoded short hairpin (Ad-sh) RNA and Ad- RNA were intradermally injected. Ad-sh*Cbs* and Ad-sh*Mtr* mice were conducted in comparison with Ad-shControl (Ad-shCtrl). A regimen of Ad-sh*Cbs* and Ad-sh*Mtr* with BLM was also carried out, accompanied by Ad-*Cbs* and Ad-*Mtr* with BLM to realize the overexpression of Hcy-catabolism related genes. Applied titer of the adenovirus was 1∗10^8^ pfu.

In exogenous Hcy supplementation models, mice were injected with Hcy alone and respectively. Gavage of folate was performed daily, and BLM and Hcy were injected subcutaneously every two days. The concentration of BLM was 5 mg/kg, low concentration of Hcy was 3 mg/kg, high concentration of Hcy was 15 mg/kg, and folate was 1.25 mg/kg. After 28 days of modeling, the skin was extracted, subjected to H&E, Masson and picrosirius red staining. Simultaneously, in situ RNA was extracted, and qPCR was conducted to detect the gene expression level.

### 2.6 Primary fibroblast culture

Freshly excised skin tissue was initially processed by removing epidermal and adipose tissues with sterile scissors. The remaining dermal tissue was then cut thoroughly and washed in PBS containing 5 % penicillin-streptomycin (Invitrogen, USA). Subsequently, the collected tissue samples underwent digestion by type I collagenase (Worthington, USA), at 37 °C with intermittent shaking for an hour. Following digestion, dissolved cells were collected, filtered with a 70 μm cell strainer. Thereafter, the cell pellets were centrifuged at 400×*g* for 10 min and then resuspended in complete medium (CM), which consisted of Dulbecco's modified Eagle's medium (DMEM; Gibco, USA), supplemented with 10 % fetal bovine serum (FBS; Hyclone, USA), and 1 % penicillin-streptomycin. The fibroblasts obtained from the tissue samples were cultured in a 37 °C incubator with 5 % CO_2_ for approximately 14 days until reaching confluence, denoted as generation 0. Subsequently, the fibroblasts were digested with 0.25 % trypsin-EDTA (Invitrogen, USA), and then seeded for further determination. Throughout the culture period, the cell medium was replaced every three days. Fibroblasts ranging from 3 to 7 generations were selected for this study.

In the knockdown and overexpression mouse fibroblasts models, adenoviruses expressing mouse *Cbs* (Ad-*Cbs*), *Mtr* (Ad-*Mtr*), *Cbs* shRNA (Ad-sh*Cbs*), and *Mtr* shRNA (Ad-sh*Mtr*), along with control adenoviruses (Ad-Ctrl and Ad-shCtrl), were procured from Sainuowei Biolabs. These adenoviruses were amplified by infecting HEK-293a cells for 48 h. Subsequently, the HEK cells were harvested and subjected to freeze–thaw cycles to break them open, and the released adenoviruses were purified using CsCl density gradient ultracentrifugation. Mouse passage 1 skin fibroblasts were cultured for one day, infected with an adenovirus at a concentration of 1∗10^9^ pfu for 2 h, and then cultured for an additional 48 h before further analysis. To achieve overexpression of Hcy-catabolism-related genes, a regimen combining Ad-sh*Cbs* and Ad-sh*Mtr* with TGF-β1 was implemented, alongside Ad-*Cbs* and Ad-*Mtr* with TGF-β1.

In the exogenous Hcy supplementation models, one group served as the control, while the other five groups were treated with varying conditions: low concentration of Hcy, high concentration of Hcy, TGF-β1, TGF-β1 with Hcy, and TGF-β1 with 5-methyltetrahydrofolic acid (5-mTHF). The concentrations used were 10 ng/ml for TGF-β1, 200 nM for low concentration Hcy, 1 μM for high concentration Hcy, and 50 μM for 5-mTHF. The effects on inflammation and fibrosis in skin fibroblasts were observed. After 24 h of treatment, total RNA and protein were extracted to assess gene and protein expression.

### 2.7 H&E and Masson staining

After treatment with formalin, human and mouse cutaneous tissues were paraffin-embedded. The thickness of the slices was 4 μm, and the slices were baked in an oven at 60 °C for 3 h. The wax was removed with xylene and dehydrated with anhydrous ethanol. Subsequently, Masson and H&E staining was applied to detect the pathological alterations and collagen deposition occurred in the skin tissues. Images were obtained by FSX100 Bio Imaging Navigator (Olympus, Tokyo, Japan).

### 2.8 Picrosirius red staining

The specimens were fixed in a 10 % formalin solution for a duration of 24 h prior to being embedded in paraffin. Subsequently, 5-μm sections were prepared and deparaffinized using xylene. The sections were then rinsed with tap water, followed by staining with 0.1 % Fast Green FCF for a period of 10 min and subsequently washed with acetic acid. Thereafter, the slides were stained with Picrosirius Red F3BA for 1 h. Following this, the slides were washed with acidified water, subjected to a dehydration process, cleared, and finally mounted. The slides, once stained with Picrosirius Red, were examined under polarized light using a microscope (DMi8, Leica, Germany) equipped with an objective lens.

### 2.9 Immunofluorescence staining

Stain 5 μm paraffin sections or frozen sections. The slides were enclosed with a blocking solution (3 % BSA and 0.2 % Triton X-100 in PBS solution) for 2 h. Subsequently, tissue and cell sections were incubated overnight at 4 °C with primary antibodies of pre-collagen (COL1A1 pre-peptide, 1:50, Thermo Fisher Scientific, USA), α-SMA (1:100, GB111364, Servicebio, Wuhan, China), Vimentin (1:100, T55134, Abmart, Shanghai, China), CBS (1:100, 14787-1-AP, Proteintech, Wuhan, China), MTR (1:100, 25896-1-AP, Proteintech, Wuhan, China), *p*-JAK2 (1:100, T56570, Abmart, Shanghai, China), and p-STAT3 (1:100, T56566, Abmart, Shanghai, China). Secondary antibodies coupled to Alexa 594 (Thermo Fisher Scientific, USA) were employed. Coverslips were mounted utilizing Vectashield with 4′,6-diamidino-2-phenylindole (DAPI). Fluorescence images were captured with FSX100 Bio Imaging Navigator (Olympus, Tokyo, Japan). For quantification, integrated density was analyzed using ImageJ Software.

### 2.10 Measurement of relative Hcy

The concentration of Hcy in skin tissue homogenates and cell lysates was quantified using the LMA772Ge 96T Hcy assay kit (magnetic bead Luminex single-plex sandwich method, Wuhan Yursheng Trading Company, China). Tissue samples were homogenized in cold PBS and centrifuged at 1,000×g for 20 minutes at 4°C. Cell lysates were prepared similarly, with the addition of protease inhibitors. Supernatants were stored at -80°C. The assay was conducted as per the manufacturer's protocol. Briefly, the 96-well plate was pre-wet with assay buffer and incubated with standards or samples, magnetic beads, and detection reagents at 37°C. After washing and incubation steps, the median fluorescence intensity (MFI) was measured using a Luminex 200 instrument. Hcy concentrations were determined by interpolating sample MFI values onto a standard curve, adjusted for dilution factors. The resultant data were subsequently analyzed comparatively within each experimental group.

### 2.11 Hydroxyproline levels

The hydroxyproline content was determined utilizing a Hydroxyproline Assay Kit (Jiancheng Biotech, Nanjing, China) in strict accordance with the manufacturer's instructions. The process commenced with the addition of alkaline hydrolysate to each 50 mg skin tissue sample, followed by incubation in a water bath at 95 °C for a duration of 20 min. Subsequently, the pH of the solution was meticulously adjusted to the neutral range of 6.0–6.8, and activated carbon was incorporated into the mixture. The optical density of the samples was then measured at a wavelength of 550 nm. The hydroxyproline concentration was subsequently calculated from the optical density values and expressed as ug/mg wet weight.

### 2.12 Quantitative real-time polymerase chain reaction (qPCR)

Total RNA was extracted from cultured fibroblasts, human or mouse skin tissue with TRIzol Reagent (Life Technologies) according to manufacturer's instructions. RNA was isolated and purified using a RNeasy Fibrous Tissue Mini Kit (Qiagen). Then, 0.5 μg of RNA was reverse transcribed to cDNA utilizing a PrimeScript RT Reagent Kit (Takara, China). qPCR was consequently performed utilizing a SYBR Prime Script qPCR Kit (Takara, China), and the reaction mixtures were incubated at 95 °C for 30 s followed by 40 cycles of 95 °C for 5 s and 60 °C for 34 s using a Light Cycler 96. 2^−ΔΔCT^ approach was employed to calculate the relative expression levels, in turn normalized to β-Actin. All primers are illustrated in Table 1. All the experiments were performed at least three times.

**Table 1:** Primers used in this study.

Type	Gene	Forward Primer	Reverse Primer
Mouse	β-Actin	5′ATATCGCTGCGCTGGTCGTC3′	5′AGGATGGCGTGAGGGAGAGC3′
	Acta2	5′GTCCCAGACATCAGGGAGTAA3′	5′TCGGATACTTCAGCGTCAGGA3′
	Col1a1	5′GCTCCTCTTAGGGGCCACT3′	5′CCACGTCTCACCATTGGGG3′
	Col3a1	5′CTGTAACATGGAAACTGGGGAAA3′	5′CCATAGCTGAACTGAAAACCACC3′
	Fn1	5′ATGTGGACCCCTCCTGATAGT3′	5′GCCCAGTGATTTCAGCAAAGG3′
	Tgfb	5′CCACCTGCAAGACCATCGAC3′	5′CTGGCGAGCCTTAGTITGGAC3′
	Cbs	5′CCAGGCACCTGTGGTCAAC3′	5′GGTCTCGTGATTGGATCTGCT3′
	Mthfr	5′CTGGGCACTGTTATCCATCCC3′	5′TCCTGCTGATAGAGGGTGGC3′
	Mtr	5′ATGATCCAGCGGTACAAACTAAG3′	5′CATCCGGTAGGCCAAGTGTTC3′
	Mtrr	5′GGACAGGCAAAGGCCATAG3′	5′ACCCGTGGTAGATACAACCAT3′
	Mat1a	5′GTGCTGGATGCTCACCTCAAG3′	5′CCACCCGCTGGTAATCAACC3′
	Il-1β	5′TGCCACCTTTIGACAGTGATG3′	5′TGATGTGCTGCTGCGAGATT3′
	Il-6	5′AGCCAGAGTCCTTCAGAGAGAT3′	5′AGAGCATIGGAAATTGGGGT3′
	Tnf	5′CCCTCACACTCAGATCATCTTCT3′	5′GCTACGACGTGGGCTACAG3′
	Ccl2	5′TTAAAAACCTGGATCGGAACCAA3′	5′GCATTAGCTTCAGATTTACGGGT3′
	Ccl5	5′GCTGCTTTGCCTACCTCTCC3′	5′TCGAGTGACAAACACGACTGC3′
	Cxcl10	5′CCAAGTGCTGCCGTCATTTTC3′	5′GGCTCGCAGGGATGATTTCAA3′
	Cx3cl1	5′ACGAAATGCGAAATCATGTGC3′	5′CTGTGTCGTCTCCAGGACAA3′
	Cxcl16	5′CCTTGTCTCTTGCGTTCTTCC3′	5′TCCAAAGTACCCTGCGGTATC3′
Human	β-Actin	5′CATGTACGTTGCTATCCAGGC3′	5′CTCCTTAATGTCACGCACGAT3′
	Acta2	5′AAAAGACAGCTACGTGGGTGA3′	5′GCCATGTTCTATCGGGTACTTC3′
	Col1a1	5′GAGGGCCAAGACGAAGACATC3′	5′ CAGATCACGTCATCGCACAAC3′
	Col3a1	5′GGAGCTGGCTACTTCTCGC3′	5′GGGAACATCCTCCTTCAACAG3′
	Fn1	5′CGGTGGCTGTCAGTCAAAG3′	5′AAACCTCGGCTTCCTCCATAA3′
	Cbs	5′GGCCAAGTGTGAGTTCTTCAA3′	5′GGCTCGATAATCGTGTCCCC3′
	Mthfr	5′GAGCGGCATGAGAGACTCC3′	5′CCGGTCAAACCTTGAGATGAG3′
	Mtr	5′AGCGGGAGAAGCTAAACGAAG3′	5′CGGTAGGCCAAGTGTTCAAGG3′
	Mtrr	5′ACAGCCCGCAAGTTTGTTAAG3′	5′CCAGTAACCCATACCGCAGG3′
	Mat1a	5′TCATGTTCACATCGGAGTCTGT3′	5′CATGCCGGTCTTGCACACT3′
	Il-1β	5′ATGATGGCTTATTACAGTGGCAA3′	5′GTCGGAGATTCGTAGCTGGA3′
	Il-6	5′ACTCACCTCTTCAGAACGAATTG3′	5′CCATCTTTGGAAGGTTCAGGTTG3′
	TNF-α	5′CCTCTCTCTAATCAGCCCTCTG3′	5′GAGGACCTGGGAGTAGATGAG3′
	Ccl2	5′CAGCCAGATGCAATCAATGCC3′	5′TGGAATCCTGAACCCACTTCT3′
	Ccl5	5′CCAGCAGTCGTCTTTGTCAC3′	5′CTCTGGGTTGGCACACACTT3′
	Cxcl10	5′GTGGCATTCAAGGAGTACCTC3′	5′TGATGGCCTTCGATTCTGGATT3′
	Cx3cl1	5′ACCACGGTGTGACGAAATG3′	5′TGTTGATAGTGGATGAGCAAAGC3′
	Cxcl16	5′CCCGCCATCGGTTCAGTTC3′	5′CCCCGAGTAAGCATGTCCAC3′

### 2.13 Western blot analysis

Cells were implanted into six-well plates and cultured until attachment. Subsequently TGF-β1, Hcy, and 5-mTHF were added correspondingly and incubated. Protein concentrations were determined with BCA Protein Assay Reagent (Thermo Fisher Scientific, USA). Extracted proteins were separated by SDS-PAGE and electro-transferred to polyvinylidene fluoride (PVDF) membranes (Bio-Rad Laboratories). After blocking with 5 % bovine serum albumin (Sigma-Aldrich), membranes were incubated with the following primary antibodies at 4 °C overnight: anti-α-SMA (1:1000, rabbit polyclonal, T55295, Abmart, Shanghai, China), anti-Fibronectin (1:1000, rabbit polyclonal, T59537, Abmart, Shanghai, China), anti-Collagen I (1:1000, rabbit polyclonal, TA7001, Abmart, Shanghai, China), anti-Collagen III (1:1000, rabbit polyclonal, T510299, Abmart, Shanghai, China), anti-JAK2 (1:1000, rabbit polyclonal, T55287, Abmart, Shanghai, China), anti-*p*-JAK2 (1:1000, rabbit polyclonal, T56570, Abmart), anti-STAT3 (1:1000, rabbit polyclonal, T55292, Abmart, Shanghai, China), anti-p-STAT3 (1:1000, rabbit polyclonal, T56566, Abmart, Shanghai, China), anti-CBS(1:5000, rabbit polyclonal, 14787-1-AP, Proteintech, Wuhan, China), anti-MTR(1:1000, rabbit polyclonal, 25896-1-AP, Proteintech, Wuhan, China), and anti-GAPDH (1:1000, mouse monoclonal, T0004; Affinity, UK). After washing, the membranes were incubated with horseradish peroxidase (HRP)-conjugated secondary antibodies (goat anti-mouse IgG, 1:5000, 115-035-003, and goat anti-rabbit IgG, 1:5000, 111-035-003; Jackson Immunoresearch, US) for 1h at room temperature, and signals were detected by enhanced chemiluminescence.

### 2.14 Quantitative and statistical analysis

The assessment of dermal thickness and immunostaining positive cells were performed in a blinded manner. Statistical significance was assessed using an unpaired, two-tailed Student's *t*-test for comparisons between two groups to evaluate dermal thickness, hydroxyproline and Hcy concentration, qPCR data, and immunofluorescence assay. The variance was estimated within each group of data. For comparisons between two groups, the equivalence of variance was confirmed utilizing an F test. For multigroup comparisons, one-way ANOVA followed by a post hoc test was used. For nonparametric data, the Mann–Whitney *U* test was used. For nonparametric, multigroup comparisons, the Kruskal–Wallis test followed by Dunn's multiple comparisons test was used. All statistical tests were performed with Prism 9 (GraphPad Software) on Win 11. Differences of (adjusted) ∗p < 0.05, ∗∗p < 0.01, ∗∗∗p < 0.001, ∗∗∗∗p < 0.0001, were considered statistically significant and they were reported in the figures and figure legends.

## 3 Results

### 3.1 RNA-sequencing analysis of publicly available datasets unveiled an association between Hcy metabolism and skin fibrosis

To elucidate the connection between aberrant metabolic pathways and skin fibrosis, we analyzed skin tissues from both healthy individuals and keloid patients using the GSE145725 dataset. This analysis yielded 350 differentially expressed genes (DEGs), including 174 up-regulated and 176 down-regulated genes (adjusted P value < 0.05 and |Log2FC| ≥ 1.5) (Fig. S1A). KEGG and GO enrichment analyses revealed that alternative signaling pathways associated with EMT, such as focal adhesion and the TGF-beta signaling pathway, were upregulated in keloids (Fig. 1A). Conversely, metabolic pathways related to Hcy, including cysteine and methionine metabolism and the one-carbon pool by folate, were downregulated in keloids (Fig. 1B). A Circos chart further illustrated the underlying genes and pathways for critical GO (Fig. 1C) and KEGG enrichment (Fig. 1D). In addition, Gene Set Enrichment Analysis (GSEA) revealed significant enrichment in collagen binding and decreased enrichment in cysteine and methionine metabolism, as well as amino acid metabolic processes in keloids (Fig. 1E–S1B). Heat maps showed decreased expression of genes involved in Hcy catabolism, including *CBS*, *MTR*, 5-methylenetetrahydrofolate homocysteine methyltransferase reductase (*MTRR*), methylenetetrahydrofolate reductase (*MTHFR*), and methionine adenosyltransferase 1a (*MAT1A*) in keloids (Fig. 1G). Furthermore, Protein-Protein Interaction (PPI) network analysis revealed significant correlations between Hcy metabolism-related proteins like CBS and MTR, and fibrosis-related proteins (Fig. 1H).

**Fig. 1:**
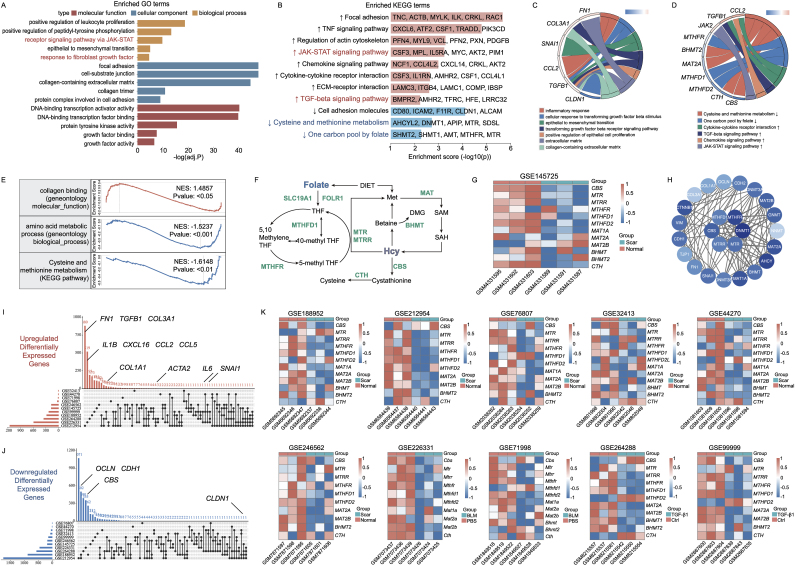
RNA-sequencing analysis unveiled impaired homocysteine (Hcy) catabolism in fibrotic skin **pathology.** (A) Enrichment analysis of GO items in GSE145725. (B) KEGG enrichment of relevant pathways. Circos plot illustrating underlying genes for significant GO terms (C) and KEGG pathways (D). (E) GSEA of keloids. (F) Schematic diagram of Hcy metabolism. (G) Heatmap depicting expression of Hcy catabolism genes, with red representing relative high expression, blue the opposite. (H) PPI network analysis between Hcy metabolism-related proteins and fibrosis-related proteins. UpSetR plot with 11 datasets overlapping homologous upregulated (I) and downregulated DEGs (J). (K) Heatmaps of other GEO databases concerning Hcy catabolism genes.

To further illustrate the decrease in Hcy metabolism-related genes in fibrotic skin, we utilized the high-throughput Gene Expression GEO database. This combined analysis included keloid datasets (GSE32413, GSE44270, GSE76807, GSE188952, GSE212954, GSE246562), bleomycin (BLM)-induced fibrotic mice skin datasets (GSE71998, GSE226331), and transforming growth factor-beta 1 (TGF-β1)-treated fibroblast datasets (GSE99999, GSE264288). In these databases, we observed a decrease in Hcy catabolism and an increase in fibrosis and inflammation in fibrotic skin. These results were visualized using the UpSetR package (Fig. 1I and J) and heat maps (Fig. 1K–S1C). Collectively, these results underscore the intense correlation between Hcy metabolism and skin fibrosis.

### 3.2 Hcy catabolism was impaired in pathogenesis of skin fibrosis

Based on bioinformatics analysis, we next investigated the expression of crucial genes involved in Hcy catabolism, specifically *CBS* and *MTR*, in the pathogenesis of skin fibrosis. We found that MTR and CBS-positive cells were reduced in human hyperplastic scar tissue through co-staining with COL1A1 (Fig. 2A, S2A). qPCR analyses confirmed elevated expression of fibrosis-related genes in HS tissue (Fig. 2B), alongside diminished levels of Hcy catabolism-related genes, such as *CBS*, *MTR*, *MTRR*, *MTHFR*, and *MAT1A* (Fig. 2C–S2B). Furthermore, elevated levels of hydroxyproline and Hcy were detected in human HS tissue (Fig. 2D and E). Notably, Hcy levels exhibited a significant correlation (p < 0.0001) with hydroxyproline levels (Fig. 2F).

**Fig. 2:**
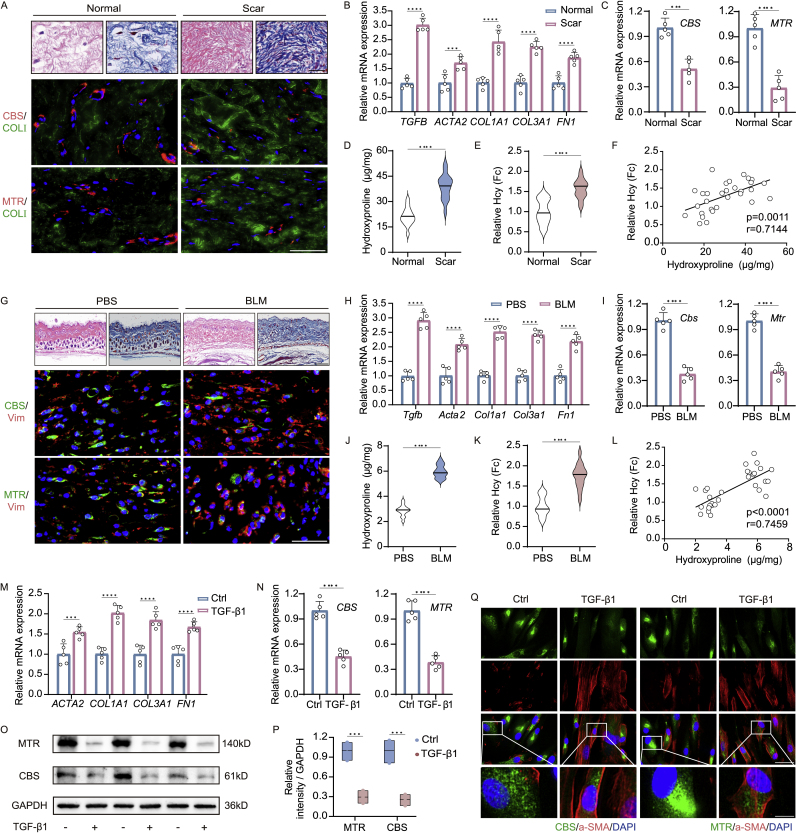
**Experimental analysis revealed Hcy accumulation due to impaired Hcy catabolism.** (A) Co-staining of CBS and MTR with COL1A1 in human hyperplastic scar tissue (scale bar, 40 μm) (n = 5), along with H&E and Masson staining (scale bar, 25 μm). qPCR analysis in human hyperplastic scar (HS) tissue of fibrosis-related genes (B) and Hcy catabolism-related genes (C) (n = 5). Hydroxyproline (D) and relative Hcy content measurement (E) (n = 15), along with correlation analysis for hydroxyproline levels (x-axis) vs. Hcy levels (y-axis) (F). (G) Co-staining of CBS and MTR with Vimentin in BLM-induced mice skin tissues (scale bar, 40 μm) (n = 5 mice), along with H&E and Masson staining (scale bar, 500 μm), qPCR analysis (H–I) (n = 5 mice), hydroxyproline (J), Hcy content (K) (n = 15 mice), and correlation analysis (L). qPCR analysis of fibrosis-related genes (M) and Hcy catabolism-related genes (N) in human skin fibroblasts exposed to TGF-β1 (n = 5). (O) Western blot analysis of MTR and CBS protein levels in TGF-β1-treated human skin fibroblasts with quantitative analysis of ratio to GAPDH (P) (n = 3). (Q) CBS and MTR co-staining with α-SMA (scale bar, 40 μm, enlarged, 12 μm) (n = 5). Results are expressed as mean ± SD. The statistical significance of differences (∗p < 0.05) was assessed by a one-way or two-way ANOVA wherever applicable, followed by Tukey's multiple-comparisons test.

In BLM-induced mice skin tissues, immunostaining and qPCR analysis also revealed reduced MTR and CBS levels and a concomitant upregulation of fibrosis genes (Fig. 2G–I, S2C, S2D). There was a notable elevation in the levels of Hcy in BLM-induced mice skin tissues, which coincided with a rise in hydroxyproline (Fig. 2J and K). Moreover, a positive correlation was observed between the levels of Hcy and hydroxyproline (Fig. 2L). Additionally, human skin fibroblasts exposed to TGF-β1 exhibited a similar trend. qPCR analyses revealed a marked reduction in Hcy catabolism-related genes (Fig. 2N–S2F). Western blot and immunostaining analyses further verified a substantial decrease in MTR and CBS protein levels in TGF-β1-treated human skin fibroblasts (Fig. 2O–Q, S2E). Collectively, these findings suggest that Hcy catabolism is impaired in the pathogenesis of skin fibrosis, leading to elevated levels of Hcy in fibrotic skin tissue.

### 3.3 CBS deficiency exacerbated skin fibrotic pathology

Next, we investigated the functions of CBS by knocking it down or overexpressing it in mouse fibroblasts and skin. In fibroblasts, the knockdown of *Cbs* using adenovirus-encoded short hairpin RNA (Ad-sh*Cbs*) was found to induce and exacerbate the upregulation of genes associated with inflammation, chemokines, and fibrosis in response to TGF-β1. Specifically, this knockdown led to increased expression of inflammatory genes such as *Il6*, *Il1b*, and *Tnfa*. Additionally, there was an upregulation of chemokine genes, including *Ccl2*, *Ccl5*, *Cxcl10*, *Cx3cl1*, and *Cxcl16*. Furthermore, genes related to fibrosis, such as *Tgfb*, *Col1a1*, *Col3a1*, *Acta2*, and *Fn1*, also showed increased expression (Fig. 3A–E, S3A-E). Intradermal injection of Ad-sh*Cbs* (Fig. S3F) markedly decreased CBS protein in skin tissue (Fig. S3G-I). Knockdown of *Cbs* exacerbated BLM-induced skin fibrosis, characterized by dermal thickening, disorganized collagen bundles, and an elevated hydroxyproline levels and COLIII/I ratio in BLM-treated mice (Fig. 3F–H). The sh*Cbs*-induced and exacerbated BLM treatments resulted in the upregulation of inflammatory and chemokine gene expression, as revealed by qPCR (Fig. 3I and J, S3J, S3K). Similarly, an increase in fibrosis-related genes was observed through qPCR and immunostaining analyses (Fig. 3K and L, S3L-N), indicating that *Cbs* knockdown in skin tissues is sufficient to induce and exacerbate skin fibrosis.

**Fig. 3:**
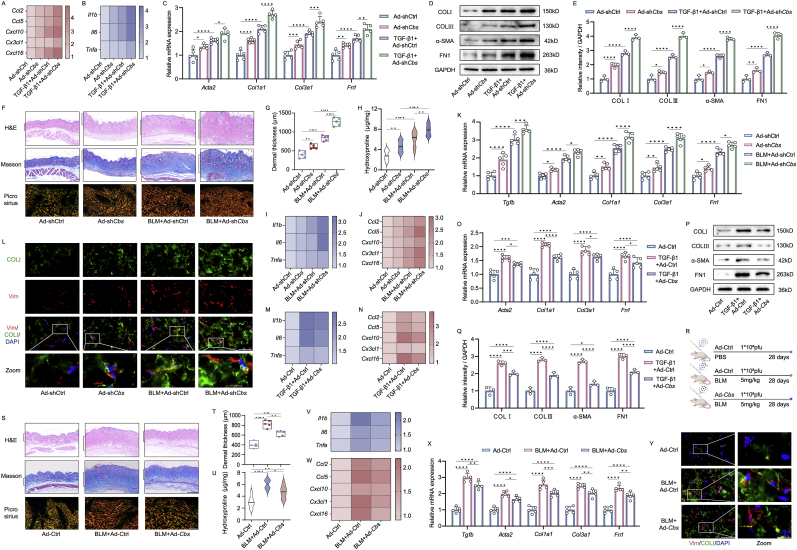
**CBS regulate pathogenesis of skin fibrosis in mice**. QPCR analysis of inflammation (A), chemokine (B) and fibrosis genes (C) (n = 5) in *Cbs* knockdown mice fibroblasts through 1∗10^9^ pfu adenovirus shRNA treatment. Western blot analysis of fibrotic proteins (D), along with quantitative analysis of ratio to GAPDH (E) in Ad-sh*Cbs* and TGF-β1 treated mice fibroblasts (n = 3). (F) H&E, Masson (scale bar, 500 μm), picrosirius red-stained images (scale bar, 100 μm), dermal thickness (G) and hydroxyproline content measurement (H) (n = 5 mice) of skin sections in *Cbs* knockdown mice. QPCR analysis of inflammation (I), chemokine (J) and fibrosis genes (K) (n = 5 mice). (L) Co-staining of Vimentin and COL1A1 (scale bar, 40 μm, enlarged, 12 μm) in *Cbs* knockdown mice (n = 5 mice). QPCR analysis of inflammation (M), chemokine (N) and fibrosis genes (O) (n = 5) in *Cbs* overexpressed mice fibroblasts through 1∗10^9^ pfu adenovirus treatment. Western blot analysis of fibrotic proteins (P), along with quantitative analysis of ratio to GAPDH (Q) in Ad-*Cbs* and TGF-β1 treated mice fibroblasts (n = 3). (R) Experimental design for the overexpression of *Cbs* and induction of skin fibrosis by BLM. Histological analysis (S), dermal thickness (T), hydroxyproline measurement (U), qPCR analysis of inflammation, chemokine and fibrosis gene (V–X), immunofluorescence co-staining of COLI and Vimentin (scale bar, 40 μm, enlarged, 12 μm) (Y) in mice subjected to BLM and intradermal injection of Ad-Ctrl or Ad-*Cbs* (n = 5 mice). Results are expressed as mean ± SD. The statistical significance of differences (∗p < 0.05) was assessed by a one-way or two-way ANOVA wherever applicable, followed by Tukey's multiple-comparisons test.

To further validate the role of CBS, we overexpressed it in mouse fibroblasts through an adenovirus-encoded *Cbs* (Ad-*Cbs*), which significantly restored CBS protein expression levels (Fig. S3O-Q). Overexpression of *Cbs* alleviated TGF-β1-induced inflammation and chemokine genes expression (Fig. 3M and N, S3R, S3S), accompanied by fibrosis phenotypes (Fig. 3O–Q). Intradermal injection of Ad-*Cbs* in mouse skin tissues significantly increased CBS protein levels (Fig. 3R and S3T-V). Histological and hydroxyproline analyses demonstrated that *Cbs* overexpression mitigated dermal thickness and collagen accumulation in BLM-induced mice (Fig. 3S–U). qPCR and immunostaining analyses also showed that *Cbs* overexpression alleviated inflammation, chemokine, and fibrosis phenotypes in BLM-induced mice (Fig. 3V–Y, S3W-AA). These results underscore the critical role of CBS in regulating skin fibrosis.

### 3.4 MTR reduction plays a critical role in skin fibrosis progression

We investigated the activity of MTR during the pathogenesis of skin fibrosis by knocking it down or overexpressing it in mouse fibroblasts and skin. Treatment with Ad-sh*Mtr* resulted in a significant decrease in MTR protein expression levels in fibroblasts (Fig. S4A-C). This reduction was associated with the activation and upregulation of inflammation-related and chemokine gene expression (Fig. 4A, 4B, S4D, S4E). Further analyses, including qPCR, western blotting, and immunostaining, provided additional confirmation of the exacerbation of fibrotic markers in fibroblasts following Ad-sh*Mtr* treatment (Fig. 4C–E). The intradermal injection of Ad-sh*Mtr* effectively reduced MTR levels in the skin within four weeks (Fig. 4F), leading to decreased MTR protein expression (Fig. S4F-H). In BLM-treated mice injected with Ad-sh*Mtr*, severe skin fibrosis was observed, characterized by dermal thickening, disorganized collagen bundles, and an elevated hydroxyproline levels and COLIII/I ratio (Fig. 4G–I). Knockdown of *Mtr* significantly increased the mRNA levels of chemokines, inflammation, and fibrosis markers, indicating that *Mtr* knockdown in skin tissues is sufficient to onset and worsen skin fibrosis (Fig. 4J–M, S4I-M).

**Fig. 4:**
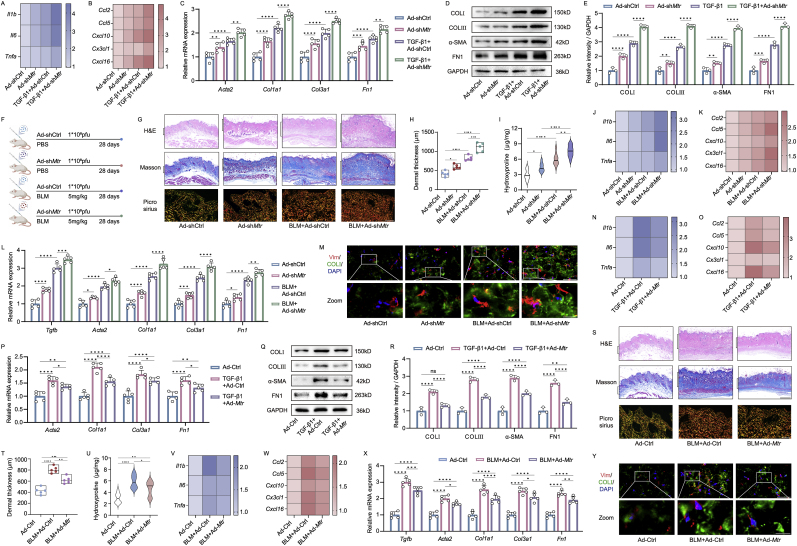
MTR deficiency exacerbated skin fibrotic pathology in mice. QPCR analysis of inflammation (A), chemokine (B) and fibrosis genes (C) (n = 5) in *Mtr* knockdown mice fibroblasts through 1∗10^9^ pfu adenovirus shRNA treatment. Western blot analysis of fibrotic proteins (D), along with quantitative analysis of ratio to GAPDH (E) in Ad-sh*Mtr* and TGF-β1 treated fibroblasts (n = 3). (F) Experimental design for the knockdown of *Mtr* and subcutaneous injection of BLM. (G) H&E, Masson (scale bar, 500 μm) and picrosirius red-stained images (scale bar, 100 μm) dermal thickness (H) and hydroxyproline content (I) in Ad-sh*Mtr* and BLM mice skin (n = 5 mice). qPCR analysis of inflammation (J), chemokine (K) and fibrosis genes (L) (n = 5 mice). (M) Co-staining of Vimentin and COL1A1 (scale bar, 40 μm) in mice subjected to BLM and intradermal injection of Ad-Ctrl or Ad-sh*Mtr* (n = 5 mice). QPCR analysis of inflammation (N), chemokine (O) and fibrosis genes (P) (n = 5) in *Mtr* overexpressed fibroblasts through 1∗10^9^ pfu adenovirus treatment. Western blot analysis of fibrotic proteins (Q), along with quantitative analysis of ratio to GAPDH (R) in Ad-*Mtr* and TGF-β1 treated fibroblasts (n = 3). Histological analysis (S), Dermal thickness (T), Hydroxyproline measurement (U), qPCR analysis of inflammation, chemokine and fibrosis gene (V–X), immunofluorescence co-staining (scale bar, 40 μm, enlarged, 12 μm) (Y) in BLM mice that had undergone intradermal injection of Ad-Ctrl or Ad-*Mtr* (n = 5 mice). Results are expressed as mean ± SD. The statistical significance of differences (∗p < 0.05) was assessed by a one-way or two-way ANOVA wherever applicable, followed by Tukey's multiple-comparisons test.

We further validated the role of MTR in fibroblasts using an adenovirus-encoded *Mtr* (Ad-*Mtr*), which significantly restored MTR protein expression levels (Fig. S4N-P). This overexpression of *Mtr* effectively alleviated TGF-β1-induced inflammation and the expression of chemokine genes (Fig. 4N and O, S4Q, S4R), and was accompanied by a reduction in fibrosis phenotypes (Fig. 4P–R). Additionally, intradermal injection of Ad-*Mtr* into mouse skin tissues markedly increased MTR protein levels in the skin (Fig. S4S-V). Consequently, overexpression of *Mtr* led to substantial reductions in BLM-induced dermal thickening, disorganized collagen bundles, the elevated COLIII/I ratio, and hydroxyproline levels (Fig. 4S–U). Furthermore, the expression of inflammation and chemokine genes in the skin was decreased in *Mtr*-overexpressing mice (Fig. 4V and W, S4W, S4X). Skin fibrosis genes expression was also modestly decreased in these mice (Fig. 4X and Y, S4Y-AA). These results suggest that MTR plays a critical role in mitigating skin pathogenesis in mouse models.

### 3.5 Hcy accumulation promoted spontaneous and BLM-induced skin fibrosis

In *Cbs* and *Mtr* knockdown mouse fibroblasts, we observed an increased level of Hcy (Fig. 5A and B). Conversely, overexpression of *Cbs* and *Mtr* reduced Hcy levels in TGF-β1-induced mouse fibroblasts (Fig. S5A, S5B). This prompted us to investigate whether Hcy accumulation is associated with skin fibrosis. We analyzed inflammation and fibrotic gene expression in fibroblasts exposed to various concentrations of Hcy (200 nM, 1 μM). Inflammation, chemokine, and fibrosis gene expression progressively increased in Hcy-stimulated human skin fibroblasts, exhibiting a dose-response relationship (Fig. S5C-H). Given the significant fibrosis-inducing interaction at low Hcy concentrations, we used 200 nM in subsequent experiments for further validation. As illustrated in Fig. 5C and D, and S5I, S5J, Hcy increased inflammation and chemokine generation in TGF-β1-treated human skin fibroblasts. This augmentation of fibrotic genes was confirmed through qPCR and Western blot analysis, as well as immunostaining of double-positive COL1A1^+^α-SMA^+^ cells in human skin fibroblasts (Fig. 5E–H, S5K).

**Fig. 5:**
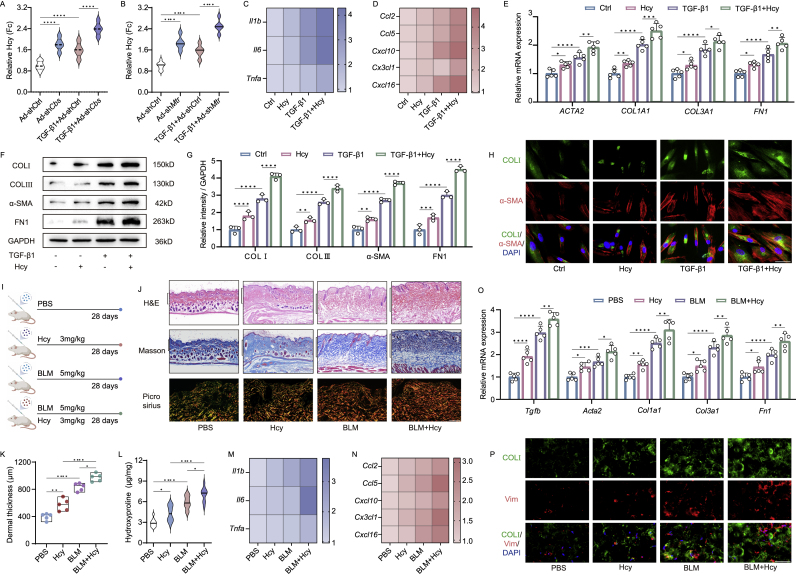
Exogenous Hcy accumulation induced and aggravates skin fibrosis. Relative Hcy concentration measurement in Ad-sh*Cbs* (A) and Ad-sh*Mtr* (B) treated fibroblasts (n = 10). qPCR analysis of inflammation (C), chemokine (D) and fibrosis genes (E) in Hcy stimulated (0, 200 nM) cells, also in comparison with TGF-β1 treated human skin fibroblasts (10 ng/ml) (n = 5). Western blot analysis of fibrotic proteins (F), along with quantitative analysis of ratio to GAPDH (G) (n = 3). (H) Immunofluorescence co-staining of COL1A1 with α-SMA with fluorescence scoring (scale bar, 40 μm) (n = 5). (I) Experimental design for fibrotic examination in subcutaneous injected Hcy and BLM mice. (J) H&E, Masson (scale bar, 500 μm) and picrosirius red-stained images (scale bar, 100 μm), dermal thickness (K) and hydroxyproline content measurement (L) (n = 5 mice) in subcutaneous injected Hcy and BLM mice. qPCR analysis of inflammation (M), chemokine (N) and fibrosis genes (O) (n = 5 mice). Immunofluorescence co-staining of COL1A1 and Vimentin (scale bar, 40 μm) (n = 5 mice) (P) in subcutaneous injected Hcy and BLM mice. Results are expressed as mean ± SD. The statistical significance of differences (∗p < 0.05) was assessed by a one-way or two-way ANOVA wherever applicable, followed by Tukey's multiple-comparisons test.

Similar phenomenon concerning Hcy concentration alterations was observed in Ad-sh*Cbs*, Ad-sh*Mtr*, Ad-*Cbs*, Ad-*Mtr* injected mice skin tissues (Fig. S5L-O). Therefore, exogenous Hcy supplementation was conducted. In vivo, subcutaneous administration of Hcy every two days over four weeks induced skin thickening in mice, demonstrating a dose-dependent response (3 mg/kg, 15 mg/kg) (Fig. S5P-R). Masson staining revealed pronounced fibrosis and collagen deposition, consistent with hydroxyproline levels (Fig. S5Q, S5S). Additionally, qPCR analyses showed a dose-dependent upregulation of inflammation, chemokine, and fibrosis genes following subcutaneous injection of Hcy, accompanied by an elevated percentage of Vimentin and COL1A1-positive cells (Fig. S5T-W). To further substantiate the adverse impacts of Hcy on fibrosis, subcutaneous Hcy and BLM injections was administered every two days for four weeks (Fig. 5I). H&E analysis and dermal thickness evaluation revealed that Hcy induced and exacerbated skin fibrotic changes (Fig. 5J and K, S5X, S5Y). Masson and picrosirius red staining corroborated these findings, along with elevated skin collage and hydroxyproline content (Fig. 5J and L, S5X, S5Z). Concurrently, qPCR analysis exhibited heightened levels of inflammation and chemokine expression in the skin of Hcy-treated mice (Fig. 5M and N, S5AB, S5AC). Immunostaining analyses further displayed a significant increase in the percentage of COL1A1-and Vimentin-positive cells (Fig. 5P–S5AD), along with elevated mRNA levels of fibrotic genes (Fig. 5O–S5AA). Collectively, these results suggest that Hcy accumulation promoted spontaneous and BLM-induced skin fibrosis.

### 3.6 Folate treatment restored Hcy catabolism and ameliorated skin fibrosis

In NASH, folate has been reported to restore the expression of Hcy metabolism genes and significantly reduce fibrosis expression [15]. High-throughput analysis of the GEO database revealed decreased expression of folate transporter receptor-related genes, such as solute carrier family 19 member 1 (*Slc19a1*) and folate receptor alpha (*Folr1*), in fibrotic skin (Fig. 6A, S6A). This finding indicates a deficiency of folate during the skin fibrosis pathology. Consequently, we investigated whether folate supplementation could rescue Hcy metabolism and fibrotic gene expression in skin fibrosis. In TGF-β1-treated human skin fibroblasts, folate supplementation significantly reversed the expression of Hcy catabolism genes (Fig. 6B–D, S6B–F) and decreased Hcy levels (Fig. 6E). Moreover, folate significantly inhibited TGF-β1-induced activation of inflammation, chemokine, and fibrosis genes (Fig. 6F–J, S6G-J).

**Fig. 6:**
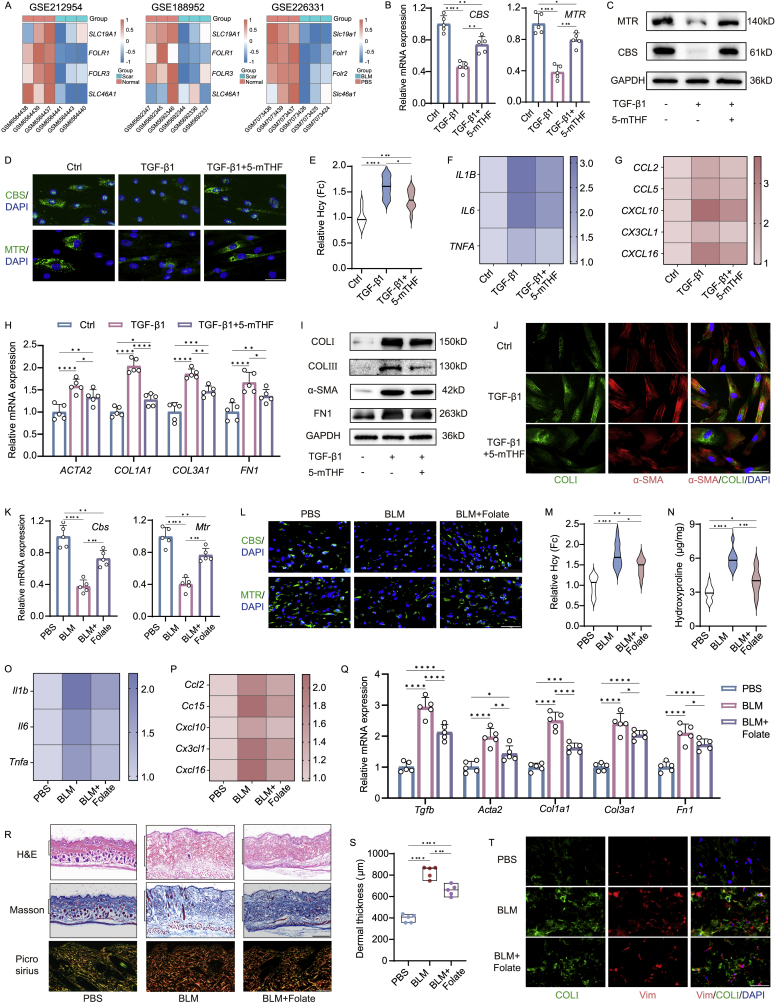
Folate treatment accelerated Hcy catabolism and ameliorated skin fibrosis. (A) Heatmaps depicting expression of folate transport genes in fibrotic skin databases. qPCR (n = 5) (B), Western blot (n = 3) (C), immunofluorescence (n = 5) (D) of CBS and MTR (scale bar, 40 μm) and relative Hcy content (n = 10) (E) in 5-mTHF-supplemented groups (50 μM) compared with TGF-β1 treated alone. qPCR analysis of chemokine, inflammation and fibrosis genes (n = 5) (F–H), Western blot analysis of fibrotic proteins (n = 3) (I), immunofluorescence co-staining of COL1A1 with α-SMA (scale bar, 40 μm) (n = 5) (J) in 5-mTHF-treated human skin fibroblasts. QPCR (K), immunofluorescence (L) of CBS and MTR (n = 5 mice), relative Hcy content (n = 10 mice) (M) in folate-supplemented mice (1.25 mg/kg, gavage) compared with BLM injected alone. (N) Hydroxyproline content measurement (n = 5 mice). qPCR analysis of inflammation, chemokine and fibrosis genes (n = 5 mice) (O–Q), H&E, Masson (scale bar, 500 μm) and picrosirius red-stained images (scale bar, 100 μm) (R), dermal thickness (S) and immunofluorescence co-staining of COL I and Vimentin (scale bar, 40 μm) (T) (n = 5 mice) in folate-supplemented mice. The statistical significance of differences (∗p < 0.05) was assessed by a one-way or two-way ANOVA wherever applicable, followed by Tukey's multiple-comparisons test.

To further confirm folate's effect on skin fibrosis, mice were subcutaneously injected with bleomycin to induce skin fibrosis and supplemented with folate gavage once daily for four weeks (Fig. S6K). Folate supplementation restored the expression of Hcy catabolism genes and markedly reduced Hcy levels in the skin of BLM-induced mice (Fig. 6K–M, S6L-N). Remarkably, folate also reduced the expression of inflammation and chemokine genes, as well as fibrosis gene expression and skin hydroxyproline levels (Fig. 6N–Q, S6O, S6P). Histological analysis and picrosirius red staining demonstrated that folate efficiently rescued dermal fibrotic thickening and collagen deposition, alleviating the fibrotic alterations induced by BLM treatment (Fig. 6R and S). Consistent with lower collagen levels, folate-treated skin exhibited significantly less fibrosis biomarkers COL1A1 and Vimentin compared to bleomycin-treated mice (Fig. 6T–S6Q). These results collectively suggest that folate supplementation restores Hcy catabolism and prevents the development of BLM-induced skin fibrosis.

### 3.7 Hcy induced skin fibrosis by promoting JAK2/STAT3 signaling pathway

To elucidate the role of Hcy in the development and progression of skin fibrosis, we investigated its potential mechanisms using bioinformatics analysis. GO, KEGG (Fig. 1A and B) and GSEA (Fig. 7A) revealed a significant correlation between the JAK2/STAT3 pathway and fibrotic skin pathologies. Consequently, we conducted experimental verification of the relationship between Hcy and the JAK2/STAT3 pathway. Our results demonstrated that the protein levels of phosphorylated JAK2 (*p*-JAK2) and phosphorylated STAT3 (p-STAT3) increased in Hcy-stimulated human skin fibroblasts in a dose-dependent manner, indicating activation of the JAK2/STAT3 pathway (Fig. 7B–D, 7K, S7A). Furthermore, combined treatment with exogenous Hcy and TGF-β1 synergistically increased expression of *p*-JAK2 and p-STAT3 levels (Fig. 7E–G, 7K, S7B). Given that folate converts Hcy to methionine, thereby reducing Hcy levels, we explored the impact of folate on TGF-β1-induced upregulation of the JAK2/STAT3 pathway. Our results found that folate effectively suppressed the activation of *p*-JAK2 and p-STAT3 induced by TGF-β1 (Fig. 7H–J, 7K, S7C). In vivo studies further supported these findings. Subcutaneous administration of Hcy over four weeks resulted in elevated *p*-JAK2 and p-STAT3 expression, as well as an increase in *p*-JAK2 and p-STAT3-positive cells compared to BLM treatment alone, suggesting that Hcy accumulation enhances JAK2/STAT3 pathway activation (Fig. 7L–S7D, S7E). Conversely, folate administration significantly decreased the percentage of *p*-JAK2 and p-STAT3 positive cells in BLM-treated mice (Fig. 7L–S7F). Collectively, these results indicate that Hcy accumulation contributes to the activation of the JAK2/STAT3 pathway, while folate supplementation mitigates this activation, providing protective effects against skin fibrosis.

**Fig. 7:**
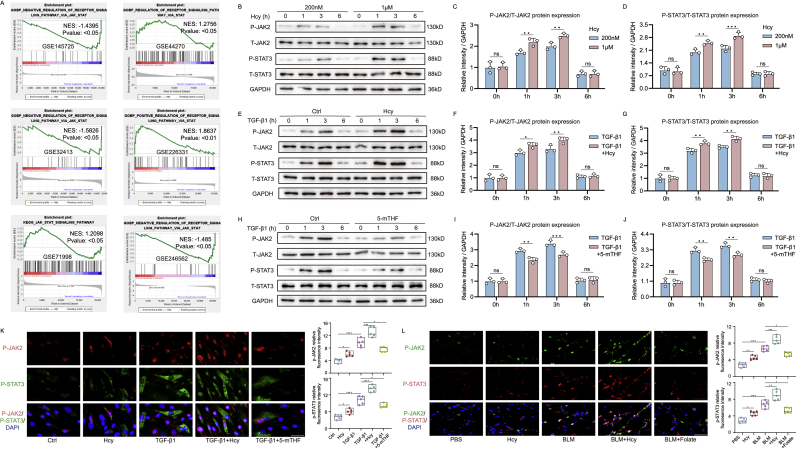
Hcy induced skin fibrosis by promoting JAK2/STAT3 signalin**g pathway.** (A) GSEA analysis of JAK2-STAT3 signaling pathway in fibrotic skin databases. (B) Western blot analysis of *p*-JAK2 and p-STAT3 protein levels in Hcy stimulated human skin fibroblasts (0, 200 nM, 1 μM). Quantitative analysis of P-JAK2/T-JAK2 (C) and P-STAT3/T-STAT3 protein levels of ratio to GAPDH (D) (n = 3). (E) Western blot analysis of *p*-JAK2 and p-STAT3 protein levels in Hcy (200 nM) combine TGF-β1 (10 ng/ml)-treated human skin fibroblasts. Quantitative analysis of P-JAK2/T-JAK2 (F) and P-STAT3/T-STAT3 protein levels of ratio to GAPDH (G) (n = 3). (H) Western blot analysis of *p*-JAK2 and p-STAT3 protein levels in TGF-β1 (10 ng/ml) and 5-mTHF-treated (50 μM) human skin fibroblasts. Quantitative analysis of P-JAK2/T-JAK2 (I) and P-STAT3/T-STAT3 protein levels of ratio to GAPDH (J) (n = 3). (K) Immunofluorescence co-staining of *p*-JAK2 with p-STAT3 with fluorescence scoring (scale bar, 40 μm) in Hcy stimulated (200 nM), TGF-β1 (10 ng/ml) and 5-mTHF (50 μM) supplemented fibroblasts (n = 5). (L) Immunofluorescence co-staining in Hcy stimulated (3 mg/kg), BLM (5 mg/kg) and folate supplemented (1.25 mg/kg) mice models (scale bar, 40 μm) (n = 5 mice). The statistical significance of differences (∗p < 0.05) was assessed by a one-way or two-way ANOVA wherever applicable, followed by Tukey's multiple-comparisons test.

## 4 Discussion

Abnormalities in Hcy levels have been linked to various fibrotic diseases, including SSc and NASH [15,18]. In this study, we demonstrate that the expression of Hcy catabolism-related genes, CBS and MTR, is decreased in fibrotic skin, leading to the accumulation of Hcy. Our gain-of-function and loss-of-function experiments in mice show that the downregulation of CBS and MTR is crucial for the pathogenesis of skin fibrosis. Furthermore, we found that the accumulation of Hcy contributes to the induction and exacerbation of skin fibrosis. Importantly, we discovered that remodeling Hcy metabolism through folate supplementation significantly ameliorated skin fibrosis in mice treated with BLM.

Previous studies have reported a positive correlation between serum Hcy levels and the SSc in patients [18]. However, it remained unclear whether this association was due to intra-skin fibroblast Hcy or systemic effects of Hcy. Through bioinformatics analyses, we discovered that Hcy catabolic genes were decreased in fibrotic skin, leading to Hcy accumulation. In BLM-induced skin fibrosis, fibroblasts converted into myofibroblasts, expressing α-SMA and inducing proliferation and migration [19]. These highly proliferative myofibroblasts secreted large amounts of ECM proteins [20]. Our study demonstrated that Hcy supplementation enhanced the expression of pro-fibrotic markers such as COL1A1, α-SMA, Fibronectin, and Vimentin both in vitro and in vivo. Consistent with our findings, previous research has shown a significant positive correlation between Hcy levels and liver and cardiac fibrosis [15,21]. Additionally, we found that knockdown of endogenous *Cbs* and *Mtr* by injecting Ad-sh*Cbs* and Ad-sh*Mtr* increased Hcy accumulation and the expression of pro-fibrotic genes. Conversely, overexpression of *Cbs* and *Mtr* promoted Hcy catabolism and reduced the expression of pro-fibrotic genes. This is consistent with the findings of Madhulika Tripathi et al., who demonstrated that knockdown of *Cbs* in the liver aggravates the progression of NASH [15]. These results indicate that impaired Hcy catabolism in fibrotic skin causes Hcy accumulation, which induces and exacerbates skin fibrosis.

Folate serves as a substrate for MTHFR, while vitamin B12 functions as a co-factor for Met synthase [22]. Together, they play critical roles in the MTHFR cycle, converting Hcy to Met [22]. Studies have reported decreased serum folate levels in fibrosis-related diseases such as hepatic fibrosis [15], pulmonary fibrosis [23], and kidney fibrosis [24]. Folate deficiency has been shown to exacerbate pro-fibrotic mediators and collagen deposition in these conditions. Madhulika Tripathi's research indicated that dietary folate supplementation promotes the enzymatic conversion of Hcy to methionine, reduces HHcy and improves hepatic histology in mice with pre-established NASH [15]. Given these findings, we investigated whether folate supplementation could restore skin Hcy metabolism and decrease skin fibrosis progression. Our results showed that folate prevented and reversed increases in skin Hcy levels and ameliorated the expression of fibrotic genes in BLM-treated mice. Folate is essential for the synthesis of S-adenosylmethionine (SAM), a universal methyl group donor required for various methylation reactions, including those involving DNA, RNA, and proteins [25,26]. DNA methylation, in particular, serves as a crucial epigenetic mechanism regulating gene expression. Tripathi et al. also demonstrated that folate enhances the enzymatic expression of Hcy metabolism, including CBS and MTR, and improves hepatic histology in mice with pre-established NASH [15]. However, the molecular mechanisms governing the regulation of CBS and MTR remain poorly understood. Given folate's role in promoting DNA methylation, it is plausible that folate restores CBS and MTR expression through epigenetic modifications. Additionally, upregulated inflammation markers IL-1β, TNF-α and IL-6 may act as matrix-preserving factors, promoting ECM deposition and increasing skin stiffness [27]. It has been reported that folate deficiency further aggravates skin inflammation and suppresses the secretion of the anti-inflammatory cytokine IL-10 [28]. Our study found that folate supplementation significantly decreased the expression of inflammatory genes *Il1b*, *Tnfa*, and *Il6* in BLM-induced mice. These results demonstrate that folate supplementation restores skin Hcy metabolism and reduces the expression of both inflammatory and fibrotic genes.

The JAK2/STAT3 signaling pathway has been consistently implicated as essential in the pathogenesis of skin fibrosis. Lim CP et al. observed elevated *p*-JAK2 and p-STAT3 levels in keloids [29]. Similarly, Xiao J et al. highlighted the aggravation of the JAK2/STAT3 pathway in peritoneal fibrotic injury [30]. TGF-β1 has been reported to trigger the activation of the JAK2/STAT3 pathway in human fibroblasts, leading to the activation of pro-fibrotic genes through nuclear translocation [31]. Our bioinformatic enrichment analysis prominently highlighted the JAK2/STAT3 signaling pathway in skin fibrosis. We also found that Hcy accumulation directly activated the JAK2/STAT3 pathway. This activation was attenuated by folate, as evidenced by our observations. Chan et al. reported that folate deficiency promoted phosphorylated STAT3 and Smad2/3, which increased fibrosis area and collagen levels in kidney tissues [32]. The positive correlation between Hcy and phosphorylated STAT3 ratio [33], as well as TGF-β1 and phosphorylated Smad2/3 ratio in renal fibrosis tissues [34], revealed the mechanisms of folate deficiency effects. Notably, strategies that block pathogenic cytokines via inhibition of the JAK2/STAT3 pathway have emerged as essential treatments for clinical conditions such as myelofibrosis [35]. These results demonstrate that folate supplementation can improve skin fibrosis by targeting Hcy-activated JAK2/STAT3 signaling.

In conclusion, our study demonstrated that Hcy accumulates in fibrotic skin due to impaired catabolism, which induces and exacerbates the progression of skin fibrosis. Folate administration promoted Hcy catabolism and improved skin fibrosis by inhibiting the JAK2/STAT3 signaling pathway. Given that the therapeutic benefits of folate in disease intervention have been increasingly documented in clinical studies, folate supplementation could be a potent therapeutic approach for skin fibrosis.

## 5 Conclusion

Our results indicate that skin fibrosis is a disease associated with Hcy metabolic disorders and suggest that targeting Hcy metabolism or supplementing folate may provide a novel strategy for skin fibrosis.

## CRediT authorship contribution statement

**Jiefeng Huang:** Writing – original draft, Project administration, Investigation. **Wuyan Lu:** Writing – original draft, Visualization, Methodology. **Shenli Zhao:** Writing – original draft, Data curation, Conceptualization. **Zixin Cai:** Writing – original draft, Software. **Linxiao Li:** Methodology, Investigation. **Zihao Hu:** Validation. **Yu Jiang:** Data curation. **Jinyi Deng:** Software. **Yiming Tang:** Formal analysis. **Chenzhang Shi:** Writing – review & editing, Funding acquisition. **Chen Wang:** Resources, Funding acquisition. **Guangpeng Liu:** Resources, Project administration. **Shuaijun Li:** Writing – review & editing, Supervision.

## Ethics approval

The study was conducted according to the guidelines of the Declaration of Helsinki and approved by the Institutional Review Board of Shanghai Ninth People's Hospital, Shanghai Jiao Tong University School of Medicine (protocol code SH9H-2021-A32-1).

## Declaration of competing interest

The authors have declared no conflict of interest.

## Figures and Tables

**figs1:**
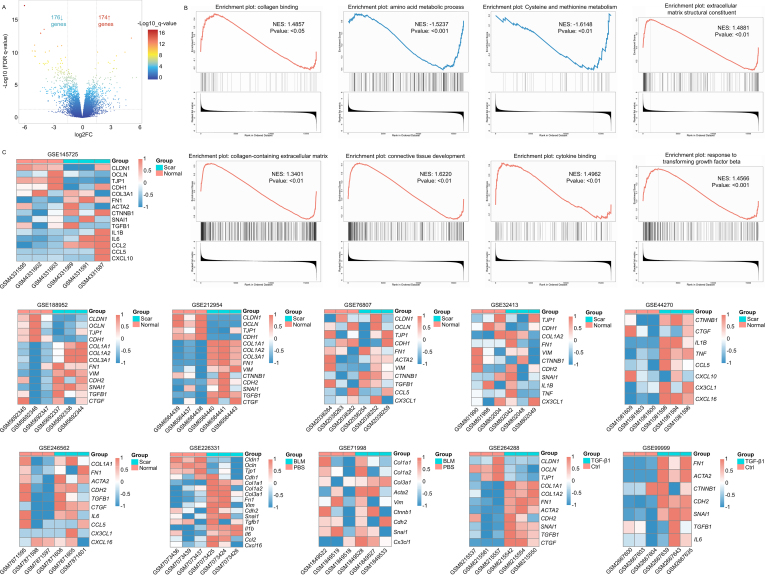


**figs2:**
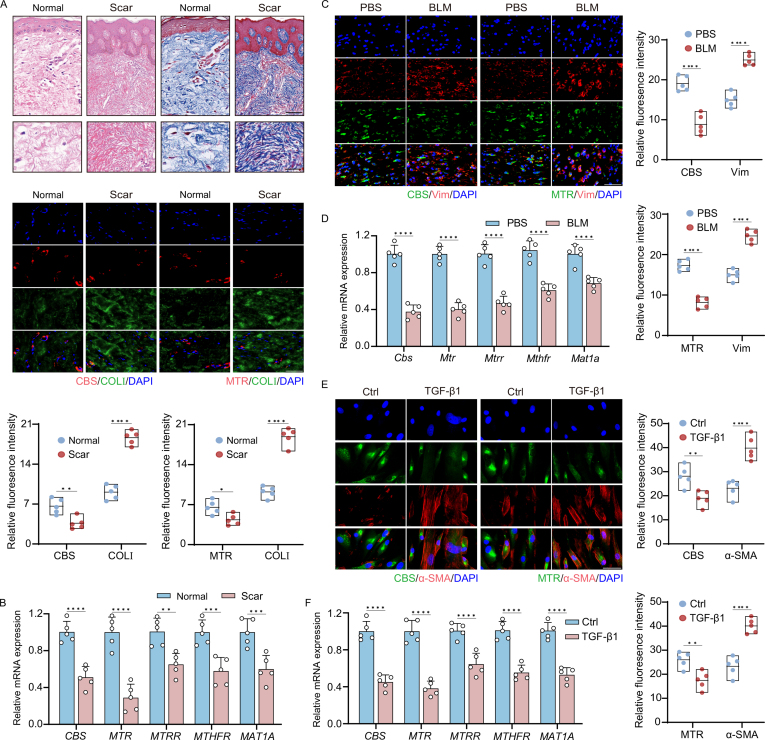


**figs3:**
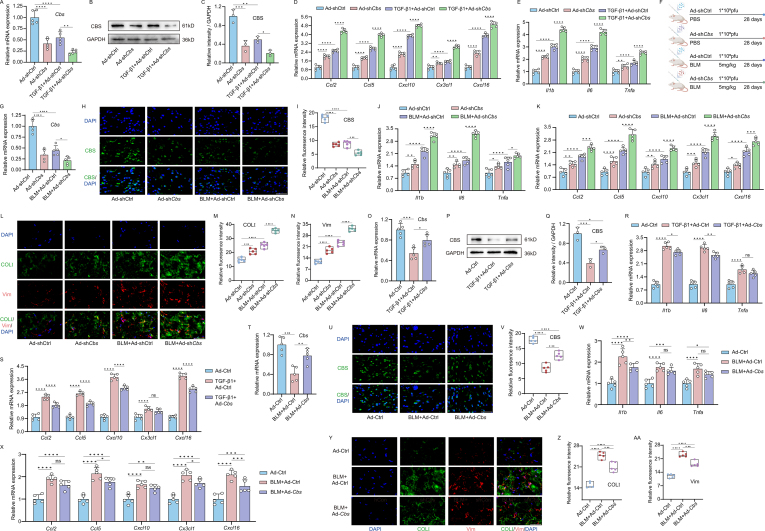


**figs4:**
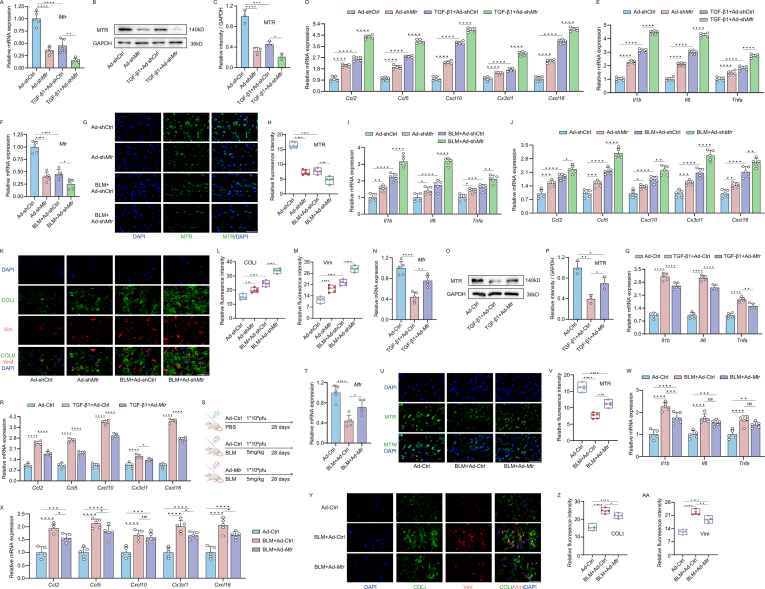


**figs5:**
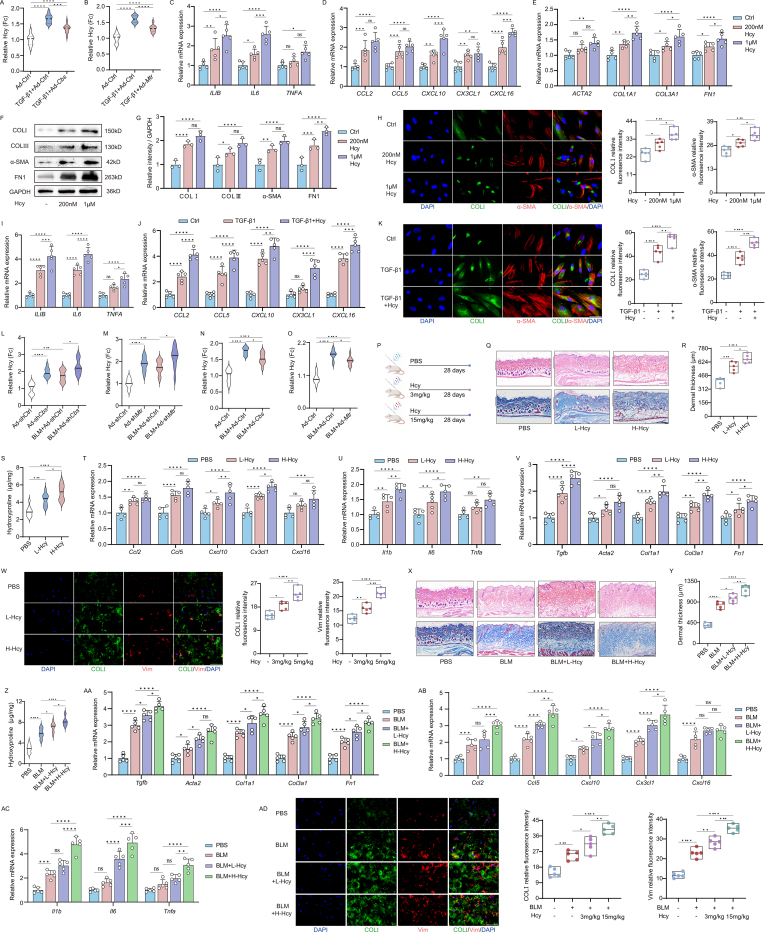


**figs6:**
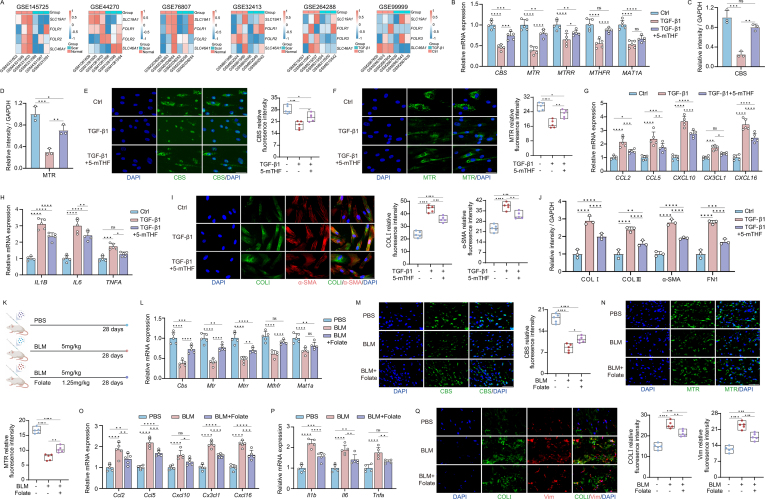


**figs7:**
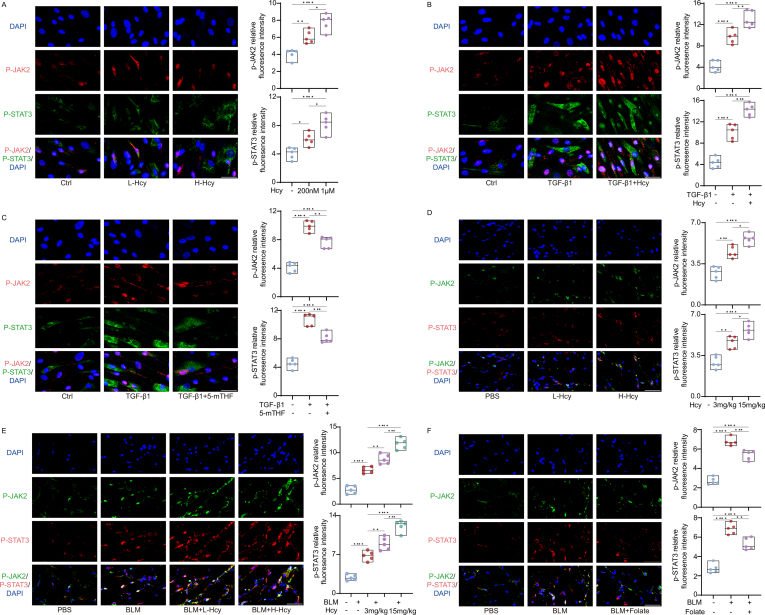


## Data Availability

We used publicly available databases and we have stated the numbers of the databases used in the article.

## Abbreviations

BLM, Bleomycin; CBS, Cystathionine β-synthase; COL1A1, Collagen type 1 α1; COL3A1, Collagen type 3 α1; ECM, Extracellular matrix; EMT, Epithelial mesenchymal transition; GAPDH, Glyceraldehyde 3-phosphate dehydrogenase; Hcy, Homocysteine; HHcy, Hyperhomocysteinemia; HS, hypertrophic scars; JAK, Janus kinase; MAT1A, Methionine adenosyltransferase I α; Met, Methionine; 5-mTHF, 5-methyltetrahydrofolic acid; MTHFD1, Methylenetetrahydrofolate dehydrogenase 1; MTHFR, Methylenetetrahydrofolate reductase; MTR, 5-methyltetrahydrofolate homocysteine methyltransferase; MTRR, 5-methylenetetrahydrofolate homocysteine s-methyltransferase reductase; PBS, Phosphate buffered saline; *p*-JAK2, Phosphorylated JAK2; p-STAT3, Phosphorylated STAT3; SAH, S-adenosylhomocysteine; qPCR, quantitative polymerase chain reaction; SAM, S-adenosylmethionine; α-SMA, α-Smooth muscle actin; SSc, Systemic sclerosis; STAT, Signal transducer and activator of transcription; TGF-β1, Transforming growth factor-β1; TNF-α, tumor necrosis factor-α.
