# Supplementary material for: Folate alleviated skin inflammation and fibrosis resulting from impaired homocysteine metabolism
Source: Redox Biol. 2025 Jan 21;80:103501. doi: 10.1016/j.redox.2025.103501 (PMC11847734; doi:10.1016/j.redox.2025.103501)
Supplement: Multimedia component 1 [file mmc1.docx]

**Compliance with Ethics Requirements**

*All procedures followed were in accordance with the ethical standards of the responsible committee on human experimentation (institutional and national) and with the Helsinki Declaration of 1975, as revised in 2008 (5). Informed consent was obtained from all patients for being included in the study.*

**Ethics approval:** The study was conducted according to the guidelines of the Declaration of Helsinki and approved by the Institutional Review Board of Shanghai Ninth People's Hospital, Shanghai Jiao Tong University School of Medicine (protocol code SH9H-2021-A32-1).

**Corresponding Author**

Chenzhang Shi, Chen Wang, Guangpeng Liu, Shuaijun Li
