# Supplementary material for: Folate alleviated skin inflammation and fibrosis resulting from impaired homocysteine metabolism
Source: Redox Biol. 2025 Jan 21;80:103501. doi: 10.1016/j.redox.2025.103501 (PMC11847734; doi:10.1016/j.redox.2025.103501)
Supplement: Multimedia component 2 [file mmc2.docx]

Supplementary Table 1. Clinical characteristics of HS patients.

| Sex | Age | Diagnosis | Sample | Site of biopsy |
| --- | --- | --- | --- | --- |
| Male | 32y | HS | HS | Earlobe |
| Female | 25y | HS | HS | Earlobe |
| Male | 37y | HS | HS | Jaw |
| Female | 48y | HS | HS | Jaw |
| Female | 51y | HS | HS | Jaw |
| Male | 36y | HS | HS | Back |
| Female | 33y | HS | HS | Back |
| Female | 49y | HS | HS | Back |
| Female | 29y | HS | HS | Back |
| Male | 34y | HS | HS | Chest |
| Male | 27y | HS | HS | Chest |
| Female | 41y | HS | HS | Chest |
| Female | 33y | HS | HS | Chest |
| Male | 48y | HS | HS | Joint |
| Female | 37y | HS | HS | Joint |
| Male | 28y | - | Healthy | Earlobe |
| Female | 31y | - | Healthy | Earlobe |
| Male | 49y | - | Healthy | Jaw |
| Male | 53y | - | Healthy | Jaw |
| Female | 33y | - | Healthy | Jaw |
| Male | 47y | - | Healthy | Back |
| Male | 56y | - | Healthy | Back |
| Female | 44y | - | Healthy | Back |
| Female | 35y | - | Healthy | Back |
| Male | 39y | - | Healthy | Chest |
| Male | 31y | - | Healthy | Chest |
| Female | 27y | - | Healthy | Chest |
| Female | 45y | - | Healthy | Chest |
| Male | 52y | - | Healthy | Joint |
| Female | 43y | - | Healthy | Joint |

HS, Hyperplastic scar.
